# Base-By-Base Version 3: New Comparative Tools for Large Virus Genomes

**DOI:** 10.3390/v10110637

**Published:** 2018-11-15

**Authors:** Shin-Lin Tu, Jeannette P. Staheli, Colum McClay, Kathleen McLeod, Timothy M. Rose, Chris Upton

**Affiliations:** 1 Biochemistry and Microbiology, University of Victoria, Victoria, BC V8W 2Y2, Canada; cindytu@uvic.ca (S.-L.T.); colummcclay@hotmail.com (C.M.); kathleen_mcleod14@hotmail.com (K.M.); 2 Center for Global Infectious Disease Research, Seattle Children’s Research Institute, Seattle, WA 98101, USA; jeannette.staheli@seattlechildrens.org (J.P.S.); trose@u.washington.edu (T.M.R.); 3 Department of Pediatrics, University of Washington, Seattle, WA 98195, USA

**Keywords:** bioinformatics, virus, comparative genomics, software, Base-By-Base, BBB, poxvirus, ASFV, MSA

## Abstract

Base-By-Base is a comprehensive tool for the creation and editing of multiple sequence alignments that is coded in Java and runs on multiple platforms. It can be used with gene and protein sequences as well as with large viral genomes, which themselves can contain gene annotations. This report describes new features added to Base-By-Base over the last 7 years. The two most significant additions are: (1) The recoding and inclusion of “consensus-degenerate hybrid oligonucleotide primers” (CODEHOP), a popular tool for the design of degenerate primers from a multiple sequence alignment of proteins; and (2) the ability to perform fuzzy searches within the columns of sequence data in multiple sequence alignments to determine the distribution of sequence variants among the sequences. The intuitive interface focuses on the presentation of results in easily understood visualizations and providing the ability to annotate the sequences in a multiple alignment with analytic and user data.

## 1. Introduction

Base-By-Base (BBB) [1,2], a multiple sequence alignment (MSA) editor, has been under development for more than 15 years and forms an integral component in the Viral Bioinformatics Resource Center (VBRC) platform (www.4virology.net) that supports comparative genomics of large DNA viruses. Although the viruses supported by VBRC are primarily poxviruses and African swine fever virus (ASFV) because of our research interests, BBB is equally valuable for other viral genomes and nucleic acid and protein sequences, which can be imported into BBB from FASTA or GenBank files. The consistent theme running throughout the development of VBRC’s tools has been to provide the virologist/biologist with easy-to-use graphical tools that let the user visualize and interact with the raw sequence data. The simplest example of this is providing the ability to quickly and visually scan a viral genome MSA for alignment errors and make manual corrections or use a second alignment algorithm to realign a section of a larger MSA. Without visually reviewing the output of a bioinformatics analysis, users may unknowingly use algorithms and parameters that are not appropriate for their analysis. For example, different tools may include or ignore gaps when calculating percent identity between sequence pairs; for example, counting a single gap of 100 nucleotides as 100 mismatches in one of a pair of sequences (1 kb alignment) that are otherwise 99% identical would create the illusion of 89% identity. The performance of a visual assessment of MSAs should be a routine “reality check” for researchers, rather than relying solely on the numerical output of alignment tools.

Previously described features of BBB include:

- Java code; uses Java Web Start to launch and automate updating of BBB for users;
- Integration with the VBRC’s viral genome database (viral orthologous clusters; VOCs);
- Alignment of sequences or subsequences (MUSCLE, ClustalW, MAFFT);
- MSA editing, with intuitive highlighting of differences between sequences;
- Applicability to gene, protein, and virus genome sequences;
- Ability to edit individual sequences;
- Intuitive graphical user interface (GUI) with ability to view sequence residues or full sequence summaries;
- Display of 6-reading frames; understands and links to gene location (data from GenBank files);
- Multiple methods to annotate sequences and MSAs, treating the BBB file as a “results notebook”.

Over the years, as we have needed novel functions in our investigations of viral genomes, we have used BBB as our standard platform for analyses that required manipulation and visual presentation of DNA and protein sequences. Thus, what otherwise might have been a solitary Perl or Python script became a feature within BBB. This enhances the functionality of BBB and provides open user access to new functions as new scripts get added. As a result, BBB has become an integrated platform with multiple features that provides a common user-friendly interface for both input of sequences and output of results.

This communication describes new features that have been incorporated into BBB: New “Advanced/Experimental tools” include j-CODEHOP, **Find Differences**, **SNIP**, and **MAFFT-add**; new “Reports” include **Get Counts**, **Get Unique Positions**, and **Get SNP Counts** (of top 2 sequences); new “Tools” that export alignment after deletion of **Specified Columns** or **Columns Containing Gap(s)**. It should be noted that these single nucleotide differences between viral genomes are not “polymorphisms” in the strictest sense, but we have used the term SNP (single nucleotide polymorphism) as it is a recognizable and understandable term among virologists.

## 2. Materials and Methods

When the BBB project began more than 15 years ago, Java was chosen as the coding language because (1) web browsers were not as capable as today’s JavaScript powered interfaces; (2) Java was the primary language taught to undergraduates at the University of Victoria; and (3) it was relatively platform-independent, promising “code once, deploy everywhere” capability, thereby eliminating the compilation and installation obstacles that hinder users that want to try out new tools.

This updated software application is available from the www.4virology.net website (previously virology.uvic.ca) and code is made available upon request under the GNU General Public License version 3. In addition, the BBB and “consensus-degenerate hybrid oligonucleotide primers” (CODEHOP) source code described below has recently been submitted to the GitHub repository (https://github.com/vbrclab/basebybase; https://github.com/vbrclab/Codehop).

j-CODEHOP can be launched from within BBB (menu: **Advanced**) or from its own web page on the www.4virology.net website. In each case, the initial step is the download of the BBB alignment editor configuration file (*.jnlp), which is started by Java Web Start on the user’s own computer by default. Java Web Start requires at least Java 7, but less than Java 11 to run. The Java Runtime Environment (JRE) can be downloaded for free, if needed.

Multiple “help” files for BBB and CODEHOP are available, including a “quick start page”, “how to doc” and “help book”, which get progressively more detailed. A j-CODEHOP tutorial is also provided. Although users are requested to register their email for use of the VBRC, this is only used to allow the resource to email users occasionally to make them aware of important new features; many users choose to use nonidentifying email addresses.

The cowpox viruses (CPXV) used as examples are: BR (AF482758.2), Norway 1994 MAN (HQ420899.1), Germany 1998 2 (HQ420897.1), Germany 1980 EP4 (HQ420895.1), Germany 2002 MKY (HQ420898.1), EleGri07/1 (KC813507.1), BeaBer04/1 (KC813491.1), RatHei09/1 (KC813504.1), GRI-90 (X94355.2), and HumGra07 (KC813510.1). The core conserved nucleotide alignment (60 kb) was used to generate a maximum-likelihood phylogenetic tree using the GTRGAMMA model in RAxML v.8.2.10 [3].

Currently, BBB has an upper limit of about 500 protein sequences (300 aa each) due to the memory assigned to the tool.

## 3. Results

### 3.1. CODEHOP Integration

**Figure 1:**
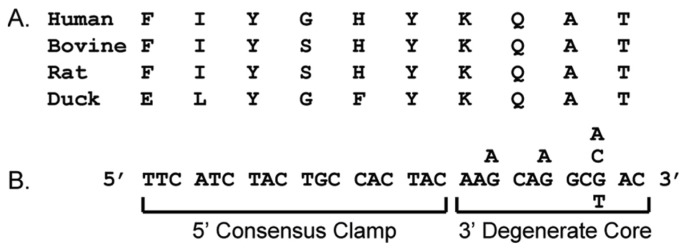
Anatomy of a “consensus-degenerate hybrid oligonucleotide primers” (CODEHOP) PCR primer. A CODEHOP is a pool of related primers containing all possible nucleotide sequences encoding 3 to 4 highly conserved amino acids within a 3′ degenerate core and a 5′ consensus clamp containing the most probable nucleotide at each position for the flanking codons. (**A**) multiple alignment of protein sequences; (**B**) predicted CODEHOP primer pool.

Even though vast amounts of genomic sequences have been obtained recently, it is unlikely that the complete genome sequence will have been determined for all living species that might provide valuable scientific and medical insights. In order to obtain sequence information for specific genes in unsequenced organisms or pathogens, a primer design strategy for PCR amplification of novel genes using ”consensus-degenerate hybrid oligonucleotide primers” (CODEHOPs) was previously developed [4]. CODEHOPs are designed from amino acid sequence motifs that are highly conserved within a gene family, and are used in PCR amplification to identify unknown related family members. Each CODEHOP consists of a pool of primers containing all possible nucleotide sequences within a 3′ degenerate core encoding a stretch of 3–4 highly conserved amino acids (Figure 1). A longer 5′ nondegenerate clamp region in the primers contains the most probable nucleotide predicted for each flanking codon. The degenerate core allows primer binding to all existing target variations in the initial PCR cycles, while the clamp region, once integrated into early PCR products, leads to efficient amplification of the PCR products in later PCR cycles. CODEHOPs designed from two adjacent conserved motifs are used to amplify the gene sequences between these motifs.

Other methods to identify unknown genes have used degenerate primers, containing most or all of the possible nucleotide sequences encoding amino acid motifs, or a consensus primer containing the most common nucleotide at each codon position within the motifs. However, unlike strictly degenerate or consensus approaches, the CODEHOP PCR approach has proven to be highly successful in amplifying distantly related genes containing significant sequence variations at low copy numbers. The primer design software and the CODEHOP PCR strategy have been utilized for the identification and characterization of new gene orthologs and paralogs in different plant, animal, and bacterial species, as well as for virus typing (e.g., enteroviruses [5]); consequently, the original publication has been cited in more than 800 subsequent publications. In addition, this approach has been successful in identifying new pathogen species and genes, as we have previously published [6,7,8,9,10,11,12].

A computer strategy to predict CODEHOP PCR primers from multiply aligned sets of related protein sequences was previously developed, which has been continuously accessible over the internet since 1998 as an integral part of the BLOCKS database developed by Steven Henikoff and hosted by the Fred Hutchinson Cancer Research Center [4]. A description of the CODEHOP program and its uses was published in the 2003 NAR Web services edition [13]. Subsequently, we developed iCODEHOP, an interactive web application independent of the BLOCKS database, to simplify and automate the process of designing CODEHOP PCR primers. The iCODEHOP program added new features, including interactive visualization of predicted CODEHOPs, phylogenetic plots for multiple aligned sequences, and user sessions on the server that allowed data to be stored during the design process [14]. However, due to advances in web browser technology, problems with the stored server sessions, and resource limitations, the iCODEHOP web application could no longer be supported and we have now developed a Java-based iteration called j-CODEHOP for integration into BBB (menu: **Advanced**).

j-CODEHOP guides users through the CODEHOP PCR primer design process, including uploading sequences, creating a multiple alignment, and identifying and visualizing primer pools that match the specified design criteria. A linked tutorial provides a step-by-step guide to demonstrate how to create CODEHOPs, using a sample FASTA file containing related sequences within the uracil DNA glycosylase family. The input to j-CODEHOP can be a set of nonaligned protein sequences or a set of aligned protein sequences. Protein sequence files may be formatted as GenBank (*.gb, *.gbk), EMBL (*.embl), BBB (*.bbb), FASTA (*.fasta, *.fas, *.fa) or CLUSTAL (*.clustal, *.clustalw). The program’s output includes a graphic showing predicted CODEHOP primers at their locations along a consensus protein sequence, a graphical representation of the region of the multiple alignment from which they are derived, and a set of metadata about each primer pool (length, degeneracy, and annealing temperature range). j-CODEHOP enables the user to visually scan the entire set of predicted CODEHOP primers to assess their relative positions and orientations within the consensus protein sequence and select individual CODEHOP primers for further analysis.

**Figure 2:**
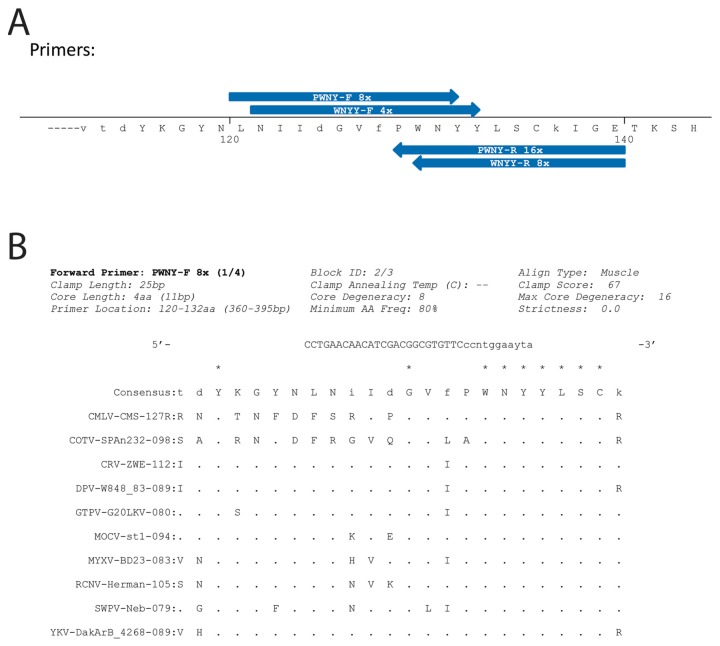
j-CODEHOP primer design output. The uracil DNA glycosylase test data set was used as input for the j-CODEHOP program and the following options were used for primer design: (1) Block making alignment tool—“MUSCLE”; (2) Codon table—“Homo sapiens”; (3) Clamp (nondegenerate 5′ region) length—“25”; (4) Core (degenerate 3′ region)—max degeneracy “16”, length of degenerate core in aa “4”, strictness (%) “0”, min aa conservation (%) “80”. Default values were used for the Advanced options: (1) 3′ nucleotide—“Invariant 3′ nt”; (2) Min block length—“5”; Primer concentration (nM)—“50”; (3) Restrict 3′ nucleotide to G or C—“unchecked”; (4) Exclude Leu, Ser, and Arg from 3′ region—“unchecked”. (**A**) Initial graphical output showing the consensus amino acid sequence for the ordered blocks of multiply aligned protein sequences. Amino acids showing conservation above the chosen minimum value are capitalized. The positions of predicted CODEHOP PCR primers are indicated showing the extent of the amino acid sequence used for primer design, the direction of the primer (forward (F) or reverse (R)), and the degeneracy of the 3′ degenerate region, i.e., 4×, 8× or 16×. A CODEHOP with 4× degeneracy is composed of a pool of 4 different primers that provide all possible sequences encoding the 3–4 highly conserved amino acid motif targeted by the CODEHOP primer. The amino acid sequence of the motif is used to name the primer, ex. “PWNY”. (**B**) The output obtained by clicking on a primer of interest in the initial graphical output, in this case primer “PWNY-F 8×”. This output shows the primer sequence (5′ to 3′), with the 5′ nondegenerate consensus region in capital letters and the 3’ degenerate region in small letters, using the international code for ambiguous nucleotides, i.e., “Y” (C,T), “R” (A,G), “N” (A,C,G,T), etc. The codons in the primer sequence are aligned with the block of multiply aligned protein sequences. Amino acid positions showing conservation above the chosen minimum are indicated with an asterisk. Amino acids within the multiple alignment which are identical to the consensus sequence are indicated with a dot. The metadata for the chosen primer design criteria are indicated, as is the primer location in amino acids and base pairs. A third panel (not shown) provides a list of primers predicted from the current amino acid block to export. The primer sequence and metadata can be exported in a “comma-separated values” (CSV) spreadsheet format. The panels shown are high-resolution representations of program output.

For the aligned protein sequences and chosen criteria, j-CODEHOP computes all primer possibilities. The initial output shows the consensus amino acid sequence for conserved blocks of the multiple protein alignment (Figure 2A). This sequence is numbered according to the positions in the multiple alignment, with capital letters for amino acids matching the minimum conservation criteria. A second window lists the possible primers to export. The user can view the consensus sequence to visualize the positions of the predicted primers, which are shown as arrows, forward or reverse. The amino acid motif targeted by the 3′ degenerate core of the primer is aligned with the primer arrow, as are the flanking amino acids specifying the 5′ non-degenerate clamp. A specific primer can be selected, which will open a third window to show the CODEHOP sequence, the block of aligned sequences used for primer design, and the primer design criteria (Figure 2B). Both forward and reverse CODEHOPs in the correct orientation need to be identified. If an insufficient number of CODEHOPs are predicted, the program can be rerun using more relaxed design criteria or the distance between the group of sequences can be reduced. Detailed methodologies have been previously published that describe the design of CODEHOP PCR primers and their use in identifying novel sequences [4,11,13,14].

### 3.2. Sequence Characteristics

**Figure 3:**
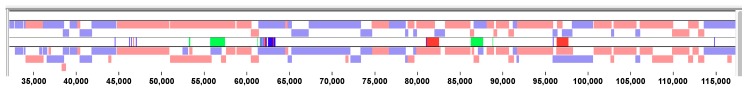
**Visual summary** from BBB. Pink and pale blue boxes represent genes transcribed to the right and left, respectively, for the genomes of two poxviruses. The centre tract indicates differences between the two sequences: Dark blue lines are SNPs (the abruptly dense SNPs turns out to be falsely assembled out-of-frame sequence from another virus) and green and red blocks show insertions and deletions (erroneously transposed sequences).

As the -omics revolution progresses, more and more researchers make use of sequence data from the various databases and, increasingly, the data behind publication claims are not presented. For example, a phylogenetic tree may be published without the MSA that was used to generate it. Given that errors in sequence naming and annotation are common in the databases, it is important that researchers check results if they are going to rely on them. Figure 3 shows a BBB **Visual Summary** of 2 virus genomes (menu: **Reports**), which are almost identical except for four large indels and a block of very poorly matching sequence. This type of visualization is a powerful tool for highlighting inconsistencies in alignments. When we further investigated these sequences (BLAST [15] searches and dotplots [16]), we found that the differences between the two genomes were entirely the result of genome assembly errors.

Additionally, under the BBB **Reports** menu, the ability to display **Sequence Similarity** and **Sequence Difference** graphs (useful for detecting recombination; not shown) has been supplemented by the plotting of a **Nucleotide Content Graph**. The user has control over which nucleotides are included in the analysis, as well as the size of the sliding window of nucleotides and the number of nucleotides that is used to “step” across the sequence. The tool also allows the user to choose which sequences from an MSA are included in these analyses. Importantly, the option to ignore gapped columns in an MSA has been included.

Additional new **Reports** features that summarize characteristics of an MSA include: (1) **Get Counts**, which counts the number of columns in the MSA with particular features, reporting the number that have a gap, a single nucleotide, two nucleotides (consensus and second type), three nucleotides, and four nucleotides; (2) **Get Unique Positions**, which lists the number of unique positions that are not gaps for each sequence; and (3) **Get SNP Counts**, which examines the top two sequences (sequences can be moved up or down within the MSA to enable sequence selection) and reports the total number of SNPs and the number of each possible substitution.

### 3.3. Counting Nucleotides Associated with Specific Sequences in MSAs

**Figure 4:**
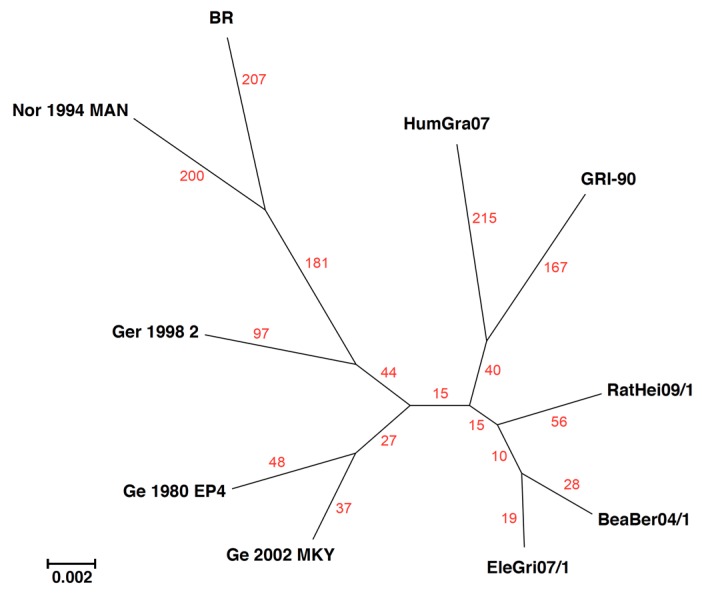
The contrast of unique SNPs found in the genomic core of 10 cowpox viruses (using the BBB **Find Differences** feature) with that of a maximum-likelihood phylogenetic tree. Red numbers denote the number of unique SNPs found for the virus that are not shared with any of the others. The phylogenetic tree branch scale denotes the average number of nucleotide substitution per site.

As noted above, the data supporting a phylogenetic tree are not often provided in manuscripts. Often, it would be useful to know the percent identity between sequences and the numbers of SNPs that distinguish one branch on a tree from another. The ability to generate a nucleotide identity matrix from an MSA is an older feature of BBB. However, now, from within the **Advanced/Experimental Tools** menu, BBB also allows a researcher to query the MSA data that support (or don’t support) a phylogenetic tree. The **Find Differences** tool can be used to count the number of SNPs that support a particular branch; e.g., “find nucleotides that are identical in sequences A, B, and C, but different in all other sequences”. Figure 4 shows the phylogenetic tree for the central relatively-conserved core (60 kb) of 10 cowpox viruses. For these sequences, the viruses in the DNA sequence identity range from 98.2–99.4%. Counting the number of SNPs unique to each sequence (red numbers in Figure 4) shows that for these cowpox sequences, the branch lengths created may not truly reflect the evolutionary distances. Instead, the lengths were likely compressed due to evidence of recombination shown in Table 1, which artificially reduced distances between distant strains.

An important feature of the **Find Differences** tool (menu: **Advanced/Experimental Tools**) is that it can allow the matching to be fuzzy. We have termed this feature “tolerance” and it can be viewed as the search “tolerating” one or more (specified by the user) sequences that do not fulfill the query. For example, the query “find nucleotides that are identical in sequences A and B but different in all other sequences, with tolerance = 1” allows any one of the sequences that should be different from A+B to be the same; different sequences are “tolerated” at different positions in the alignment. The software also: (1) Creates a list of all the positions in the alignment that satisfy the query and displays the “tolerated sequence” name if there is one, and (2) displays the distribution of SNPs in the MSA.

These BBB features were created to characterize recombination events among the poxviruses by highlighting the positions of shared SNPs. In any MSA, there will always be coincident SNPs from random events. However, for these cowpox sequences, when “nucleotides that are identical in sequences A, B, and C, but different in all other sequences” are located, some are, as expected, associated with the closest related sequence, but others are from more distant relatives. In addition, many of these coincident SNPs are found to be in nonrandom blocks, suggesting that the arrangements result from recombination among the genomes. Table 1 shows SNPs present only in CPXV-BR and CPXV-Nor1994MAN and one other sequence taken from the tree shown in Figure 4. In several instances, the common SNPs are unexpectedly clustered (Table 1) and likely result from recombination events. The results with CPXV-Ge1980EP4 and CPXV-Ge2002MKY (which are very similar (Figure 4)) as the extra sequence are dramatic; despite their similarity, CPXV-Ge1980EP4 has many more SNPs in common with the other two sequences (Table 1; 33 SNPs) than with CPXV-Ge2002MKY (Figure 4; 3 SNPs).

### 3.4. Manipulation of Sequences

As previously reported, BBB allows the addition or removal of sequences to an alignment and the removal of columns in an MSA that contains all gap characters which are often generated when removing sequences from an alignment. However, when visually inspecting the relationships between the sequences, it can also be useful to simplify the variation by removing any column that contains a gap character (menu: **Tools/Delete Columns Containing Gap(s) and Export**). Since this action will modify the sequences in use, by deleting residues from some sequences, the program will export the resulting sequence into a new BBB window and prompt the user to enter a new filename. If the sequences in an MSA are very diverged, each will have a relatively large number of unique SNPs. Since these can obscure patterns present among the SNPs shared by subsets of sequences, we also created the **SNIP** feature (menu: **Advanced/Experimental Tools**) that modifies the sequences such that the SNPs that are present only in a single sequence are changed to the consensus nucleotide. Again, because this procedure modifies the actual sequences, users are asked to save the result in a new alignment file.

When using large viral genomes and closely related viruses, SNPs may be relatively infrequent. Therefore, we incorporated a feature into BBB that allows the user to remove any specified column of nucleotides within an MSA. By removing the columns that only contain a single nucleotide, the variation is compressed into a much smaller sequence space and is more easily visualized by the user. First, the **Find Differences** tool (menu: **Advanced/Experimental Tools**) is used to find the columns that are identical (i.e., have no SNPs), then the “Search Log” is used to “List SNP Positions” only. Subsequently, these position values can then be used to delete specified columns (menu: **Tools/Delete Specified Columns and Export**).

### 3.5. Alignment of Sequences

The options for aligning complete or selected regions of sequences have been updated. Clustal Omega [17] has replaced the option to use ClustalW. Clustal Omega and MUSCLE [18] serve as options to align protein and gene length nucleotide sequences. For the alignment of large viral genomes, MAFFT [19] is the tool of choice. However, the growing number of complete genomes sequenced has translated into a more frequent need to generate larger MSAs, often to update phylogenetic trees. Although MAFFT is available at various web resources and can be easily installed on desktop computers, most users prefer to use MAFFT within BBB (menu: **Tools/Align Selection**). Therefore, we have incorporated the **MAFFT-add** option into the BBB (menu: **Advanced/Experimental Tools**) [20]. This feature allows users to align one or more new sequences to an existing alignment, which significantly reduces the compute time. For example, the alignment of 10 cowpox virus genomes takes approximately 8 min, whereas aligning one new sequence to an alignment of 9 takes a little over 1 min. The **MAFFT-add** function is also useful for scaffolding new contig sequences against a close reference sequence in the process of genome assembly.

## 4. Discussion

BBB is a foundational program of the Viral Bioinformatics Resource Centre that allows both the viewing and editing of MSAs. It has been developed over many years and serves as a platform for the comparison of large viral genomes, but it is equally useful for small DNA and RNA viral genomes, as well as gene and protein sequence alignments. The data visualization features that it provides are key to its value. These include highlighting differences between sequences in an MSA, plotting graphs of sequence similarity, adding user-comments or primers to sequence regions, and displaying forward and reverse reading frames as well as results of various sequence searches. Importantly, while BBB has provided a common interface to multiple analyses for users, it has also given programmers a standardized data input process and a single visualization canvas. One of the key features of BBB is the ability to read GenBank files so that it becomes aware of the complete set of annotations for the genomes of large viruses, allowing it to display the complete set of annotations for any large virus genome which is extremely useful for comparisons of gene features and single nucleotides.

Here, we have described a significant number of upgrades to BBB that increase the utility of the tool when working with genes, proteins or genomes. The inclusion of j-CODEHOP maintains a functioning version of the CODEHOP algorithm, which otherwise would have been lost to the research community. CODEHOP is a natural fit for BBB, since it is often used to discover novel members of viral families, but also since the generation of the protein MSAs that are used as input by j-CODEHOP to generate CODEHOP primer sequences are already an integral part of BBB. The new plots for similarity are useful for a user looking for recombination events and comparing how different genes are conserved to different degrees. Fuzzy searches for MSA columns that support or fail to support particular phylogenetic relationships bring a new process to the screening of viral genomes for small regions that have been exchanged among viruses. Following the identification of particular MSA nucleotide columns, new editing features in BBB now allow the user to manipulate these MSA columns, thereby simplifying the visualization for the user.

A variety of other tools exist to manipulate and characterize MSAs, including Jalview [21] (primarily for proteins), and IVisTMSA [22] and AliView [23] (primarily for large sequence sets). However, BBB has multiple unique features, including those described here, which make it a valuable multipurpose bioinformatics tool, especially for the comparison and characterization of viral genomes and more.

## Figures and Tables

**Table 1:** SNPs shared by CPXV-BR, CPXV-Nor1994MAN, and strain noted in the table; all other viruses in Figure 4 have a different nucleotide. SNPs close together are grouped on a single line in the table.

+BeaBer04/1
22,518, 22,519, 22,583
31,870
+RatHei09/1
4677, 4679
4886, 4896, 4899, 4917
9401
16,480
19,731
31,573
35,003
40,781
+Ge 1980 EP4
1204
10,615, 10,618
10,731, 10,747
14,442, 14,457, 14,460, 14,553, 14,574, 14,664, 14,667
15,071, 15,072, 15,076, 15,138, 15,161, 15,163, 15,171
19,409
25,381
30,528, 30,534, 30,547, 30,549, 30,556
32,797, 32,799
35,713, 35,758, 35,812
36,217
41,120
+Ge 2002 MKY,
19,510
47,392
